# Brain atrophy and cholinergic denervation in progressive supranuclear palsy: an MRI and [^18^F]-FEOBV PET study

**DOI:** 10.3389/fnins.2025.1695541

**Published:** 2025-12-03

**Authors:** Prabesh Kanel, Stiven Roytman, Giulia Carli, Robert Vangel, Jaimie Barr, Fotini Michalakis, Christopher Chauncey Spears, Peter J. H. Scott, Roger L. Albin, Nicolaas I. Bohnen

**Affiliations:** 1 Department of Radiology, University of Michigan, Ann Arbor, MI, United States; 2 Morris K. Udall Center of Excellence for Parkinson's Disease Research, University of Michigan, Ann Arbor, MI, United States; 3 Parkinson’s Foundation Research Center of Excellence, University of Michigan, Ann Arbor, MI, United States; 4 Department of Neurology, University of Michigan, Ann Arbor, MI, United States; 5 Neurology Service and GRECC, Veterans Administration Ann Arbor Healthcare System, Ann Arbor, MI, United States

**Keywords:** progressive supranucelar palsy, MRPI, FEOBV, PET, midbrain

## Abstract

**Introduction:**

Progressive supranuclear palsy (PSP) is a neurodegenerative disorder characterized by a wide spectrum of motor and cognitive impairments. Structural brain imaging biomarkers, such as the Magnetic Resonance Parkinsonism Index 2.0 (MRPI 2.0), are increasingly recognized as means of supporting a PSP diagnosis. These anatomic measures lack neurobiological correlates. In the present work, we investigated whether structural brain changes captured by MRPI 2.0 associate with whole-brain and regional cholinergic nerve terminal deficits in PSP.

**Methods:**

Structural magnetic resonance (MR) and vesicular acetylcholine transporter (VAChT) [^18^F]-fluoroethoxybenzovesamicol ([^18^F]-FEOBV) PET imaging was obtained in a sample of 16 PSP subjects. MRPI 2.0 index was quantified and correlated with whole brain VAChT binding using statistical parametric mapping (SPM), after adjustment for sex, dopaminergic medication dose and disease duration. *Post hoc* multiple regression analyses were performed to correlate regional mean VAChT binding with MRPI 2.0 (after adjusting for the same covariates), characterizing the proportion of variance in cholinergic terminal deficits explained by this structural index.

**Results:**

Voxel-wise SPM analyses revealed that MRPI 2.0 is significantly associated with cholinergic nerve terminal loss in brainstem, especially pontomesencephalic, limbic structures, insula, basal ganglia, thalamus, and cerebellar regions. *Post hoc* multiple regression analysis demonstrated that MRPI 2.0 accounts for about half of the variance in cholinergic nerve terminal integrity within relevant brain structures in PSP, showing the most robust association in the thalamus.

**Discussion:**

Structural brain changes associated with higher MRPI 2.0 index scores may be a reliable proxy measure of cholinergic terminal deficits in PSP, predominantly those in subcortical regions. Differential association of subcortical brain region cholinergic deficits with PSP-specific structural brain changes may reflect the characteristic pattern of 4R-tau pathology observed in PSP.

## Introduction

Progressive supranuclear palsy (PSP) is a debilitating neurodegenerative disorder characterized by a wide range of motor and cognitive impairments (Xie et al., 2015; Hirsch et al., 1987). These include postural instability, vertical supranuclear gaze palsy (VSGP), and executive dysfunction (Xie et al., 2015). The primary pathology involves the accumulation of hyperphosphorylated 4R-tau protein, which leads to neuronal loss and brain atrophy, particularly in the brainstem and basal ganglia (Kovacs et al., 2020; Warren et al., 2005). There is growing evidence that the cognitive and motor deficits in PSP are linked to disruption of cholinergic systems (Warren et al., 2005; Kanel et al., 2022; Gilman et al., 2010), which play critical roles in mediating attention, memory, and gait (Bohnen et al., 2021). Post-mortem studies confirmed substantial loss of cholinergic neurons in the brainstem, striatum, and basal forebrain of PSP subjects (Warren et al., 2005; Kasashima and Oda, 2003; Shinotoh et al., 1999; Pisani et al., 2007). Application of the spatially resolute positron emission tomography (PET) tracer [^18^F]-fluoroethoxybenzovesamicol ([^18^F]-FEOBV), which targets the vesicular acetylcholine transporter (VAChT), enabled quantification of high density cholinergic terminal areas, such as the striatum, cerebellum, and thalamus, that was previously limited with prior cholinesterase ligands (Kanel et al., 2022; Kanel et al., 2022; Bohnen et al., 2019). This has resulted in novel *in vivo* insights into cholinergic system changes in Alzheimer’s disease (Aghourian et al., 2017), Parkinson disease (PD) (Bohnen et al., 2025), and PSP (Kanel et al., 2022). Prior findings from our group suggested that PSP is associated with a distinctive pattern of cholinergic terminal deficits as compared to PD, with greater vulnerability of thalamic, tectal, striatal, limbic, and frontal cortical terminals (Kanel et al., 2022).

Magnetic Resonance Parkinsonism Index 2.0 (MRPI 2.0) emerged as a reliable tool for identifying structural changes associated with PSP (Quattrone et al., 2018), relying on clinical-quality MR structural images for quantification. The MRPI 2.0 is derived as a composite measure of structural changes observed in PSP, incorporating the mid-sagittal cross-sectional area of the pons and midbrain, the diameter of the superior and middle cerebellar peduncles, the maximal left-to-right width of the lateral ventricle frontal horns, and the width of the third ventricle. The anatomical regions associated with regional atrophy that contribute to MRPI 2.0 calculation, particularly the pontomesencephalic tegmentum, also include the primary locations of brainstem cholinergic nuclei (Warren et al., 2005; Quattrone et al., 2018). These cholinergic nuclei send highly collateralized projections to multiple brainstem targets, the thalamus, and regions of the basal ganglia. Through projections to the nucleus basalis of Meynert, these brainstem cholinergic nuclei may also exert influence over widespread cortical areas (Warren et al., 2005; Kanel et al., 2022; Huerta-Ocampo et al., 2020; Schofield et al., 2011). The direct relationship between this specific marker of structural atrophy and topographic loss of cholinergic innervation is unexplored.

This study aimed to investigate associations between MRPI 2.0 values and regional uptake of [^18^F]-FEOBV to determine if this established structural marker can predict the pattern and extent of cholinergic terminal deficits in PSP. We tested the hypothesis that MRPI 2.0 values are associated with the regional density of cholinergic terminals in brain regions predominantly innervated by the midbrain cholinergic nuclei, including the pedunculopontine nucleus (Ch5), the laterodorsal tegmental nucleus (Ch6) and striatal cholinergic interneurons, as measured by [^18^F]-FEOBV PET.

## Methods

### Study design and participants

Sixteen participants with PSP were recruited from the Atypical Parkinsonism Clinic at Michigan Medicine. Diagnosis was based on the 2017 MDS-PSP clinical diagnostic criteria (Hoglinger et al., 2017). All included participants showed no evidence of major vessel stroke or other intracranial lesions on MRI. This study was approved by the Institutional Review Board at the University of Michigan. Written informed consent was obtained from all participants or their legal representatives. Eight participants from the current study previously participated in studies of cholinergic deficits in PSP versus PD (Kanel et al., 2022).

### Imaging acquisition and pre-processing

MRI was performed on a 3 Tesla Philips Achieva system. PET imaging was conducted using Biograph 6 TruPoint PET/CT scanners for 13 patients and using Siemens ECAT Exact HR + scanner for 3 patients. [^18^F]-FEOBV imaging was performed as a 30-min dynamic acquisition, following a 3-h delay after intravenous tracer injection. To generate distribution volume ratio (DVR) parametric PET images, the PET imaging data were normalized using the mean activity from the eroded supratentorial white matter (reference region) (Bohnen et al., 2025). Each subject’s structural MRI images were used to create a reference region mask within the PET space that had previously been registered to the individual subject’s PET image. Motion artifacts were corrected by co-registering frames 2–6 of the PET images, which were then averaged for analysis before generating DVR parametric images. Ultimately, the parametric images were generated by normalizing each PET voxel’s value against the mean activity of the reference region. After acquiring the parametric image, the Müller-Gartner method was used to correct for partial volume effects (Müller-Gärtner et al., 1992) creating partial volume-corrected (PVC) parametric images. Structural MR images were registered and spatially normalized to the Montreal Neurological Institute (MNI) template using SPM12. The parametric PET images (PVC and non-PVC) were also normalized to the MNI space for voxel-wise analysis using the deformation field. Eight mm smoothing was applied to reduce noise.

### MRPI 2.0 calculation

The original MRPI was calculated by multiplying the ratio of the pons area to the midbrain area by the ratio of the width of the middle cerebellar peduncle (MCP) to the width of the superior cerebellar peduncle (SCP). To obtain MRPI 2.0 values, the initial MRPI value is multiplied by the ratio of the average width of the third ventricle (V3) to the maximum left-to-right width of the frontal horn (FH) of the lateral ventricles. Methods to obtain MRPI are described in detail by Quattrone et al. (2018). Visualization of the obtained measurements is provided in detail in Supplementary section S1. Following Quattrone et al. (2018), the cutoff value of MRPI 2.0 was set at 2.5 for PSP-Richardson’s syndrome (PSP-RS), 2.91 for PSP-Parkinsonism (PSP-P) patients with vertical supranuclear gaze palsy (O1 level) and 2.18 for PSP-P with slowness of vertical saccades (O2 level).

### FEOBV analysis

To investigate regional cholinergic deficits related to MRPI 2.0, we used SPM12 for a whole brain voxel-wise regression analysis without predefined hypotheses. MRPI 2.0 scores were the independent variable, and [^18^F]-FEOBV parametric images were the dependent variable, with sex, disease duration (years from symptom onset), and levodopa equivalent daily dose (LEDD) included as nuisance covariates. We applied a voxel-wise uncorrected threshold of *p* < 0.01 and a cluster-level family-wise error (FWE) correction, considering clusters with *p* < 0.05 as significant. The primary analysis presented is corrected for partial volume effects, supplemented by an analysis without correction (Supplementary section 1).

A *post hoc* multiple regression model analysis was performed, correlating mean DVR of significant cluster voxels from the voxel-wise correlation with MRPI 2.0 score, after adjustment for disease duration, LEDD, and sex to obtain an estimate on proportion of the variance in [^18^F]-FEOBV uptake within relevant regions explained by structural brain changes related to PSP. Voxel cluster DVRs were obtained by extracting the mean [^18^F]-FEOBV uptake value from spatially normalized individual images, using a mask of all the statistically significant voxels from the SPM analysis as the volume of interest (VOI). Additional *post hoc* analyses were performed in the same manner for PET-native VOI mean DVR, informed by the results of the voxel-wise correlation analysis and *a priori* knowledge about the sources of direct and indirect cholinergic projections along with their targets: thalamus, insula, hippocampus, basal forebrain, cerebellum gray matter, and whole cortex. PET-native space regional mean DVR values were extracted by applying a rigid body transform to *FreeSurfer* standard segmentation labels, bringing them into [^18^F]-FEOBV space, where the mean of all voxels belonging to each selected VOI was extracted.

All examined associations were plotted as scatterplots with lines of best fit, with regional mean DVR value (residualized with respect to LEDD and disease duration) on the Y-axis and MRPI 2.0 scores on the X-axis. All plotting was performed using the Python programming language open-source *Seaborn and Matplotlib* modules. All statistical analyses were performed in the *R* programming language using *Tidyverse* open-source package for data manipulation. Normality of residuals and homoscedasticity assumptions of all *post hoc* multiple regression models were examined using Shapiro test and Breusch-Pagan tests as implemented in *R* core and *lmtest* open-source libraries, respectively. *sjPlot* open-source *R* package was used to generate the multiple regression table.

### *Post hoc* regional volume analysis

To better elucidate the correlation between regional structural integrity and the MRPI 2.0 index in PSP, an additional *post hoc* analysis was performed. Regional gray volumes obtained via *FreeSurfer* were normalized by intracranial volume and correlated with MRPI 2.0 scores. The resulting univariate correlation coefficients were plotted over the brain using the *ggseg* open-source *R* package. Findings are presented in Supplementary material.

## Results

### Demographics

This cross-sectional study involved 16 PSP subjects (11 males and five females; mean age of 71.44 ± 4.68 years, range 63 to 79 years). All 16 subjects had probable PSP. Subtype analysis indicated that 13 subjects were classified as probable PSP-RS and one subject as probable PSP-P. Two subjects were classified as probable PSP of uncertain subtype (PSP-unk) because information on fall history was unavailable. The mean score for MDS-UPDRS Part III in the medication “off” state was 45.875 ± 11.53, and the mean modified Hoehn and Yahr stage was 3.59 ± 1.13. Using the Quattrone et al. cutoff scores, 12 subjects met MRPI 2.0 criteria for PSP. There were three probable PSP-RS cases and one probable PSP-unk case below the cutoff. See Table 1 for additional demographic information.

**Table 1:** Demographic information.

Demographics	Mean (SD)
Age	71.44 (4.68)
Gender	Females: 5 Males: 11
Disease duration (years from first symptom)	5.75 (3.40)
LEDD (mg)	303.13 (309.01)
Motor assessments	
Hoehn and Yahr stage	3.59 (1.13)
MDS-UPDRS part III	45.88 (11.53)
PSP rating scale*	35.22 (10.81)
Neuropsychological assessments	
MoCA	22.38 (5.57)

*Only available for nine subjects.

### Voxel-wise analysis

**Figure 1:**
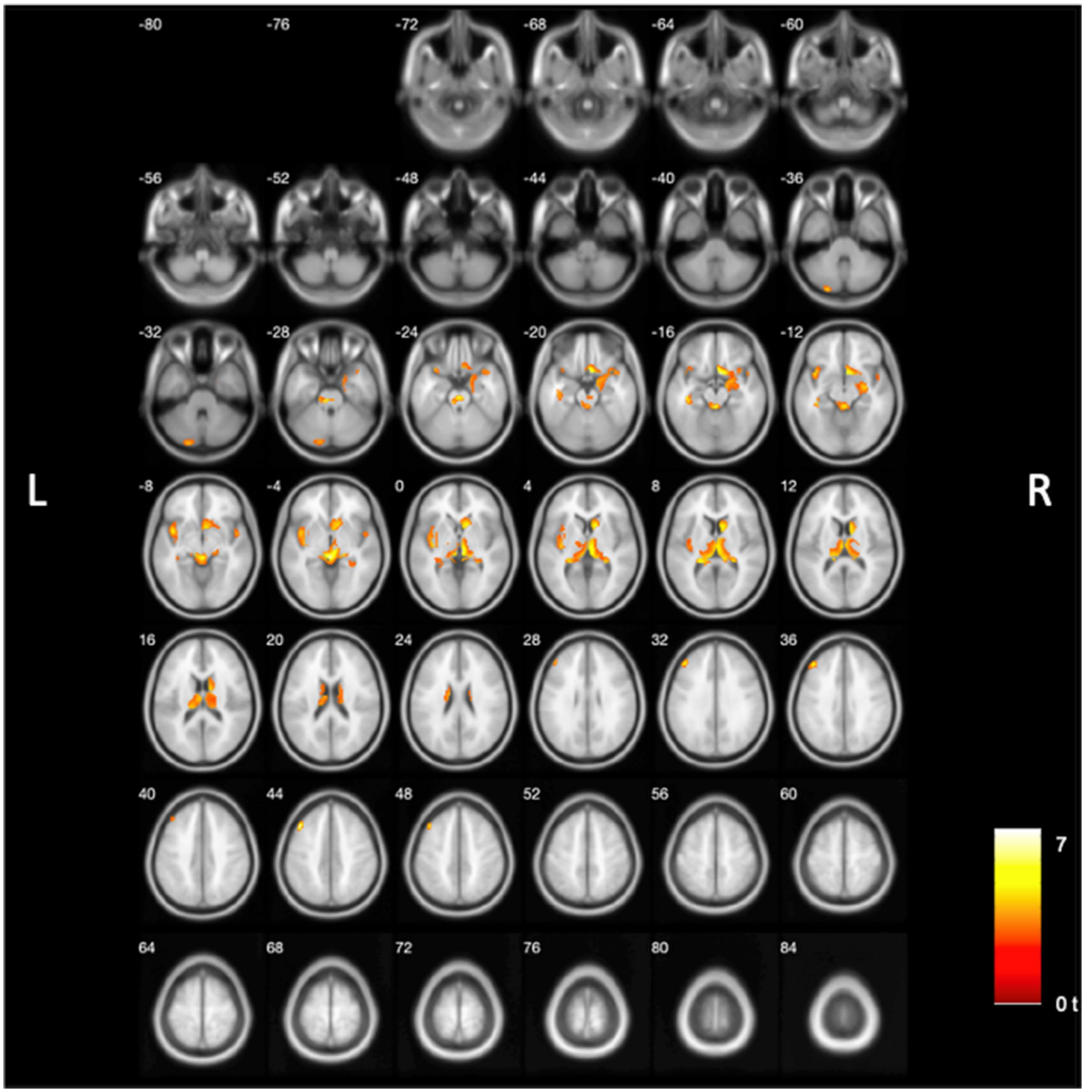
Voxel-based regression analysis of PVC corrected [^18^F]-FEOBV PET images revealed clusters of reduced cholinergic binding correlated with MRPI 2.0. Results were thresholded at uncorrected *p* < 0.01 and corrected for multiple comparisons using cluster-wise FWE-correction of *p* < 0.05. Sex, disease duration, and LEDD were covariates. Reduced binding was found in pontomesencephalic regions, the right caudate nucleus and tail, right cholinergic forebrain, right hippocampus, insula, right parahippocampal gyrus, amygdala, putamen, anterior-intralaminar- lateral posterior- mediodorsal—pulvinar—ventral nuclei of thalamus, including the metathalamus, and cerebellum.

Voxel-wise results from the SPM based voxel-based analysis indicated that lower binding of [^18^F]-FEOBV in several brain regions (Figure 1) was correlated with higher MRPI 2.0. These associations included the right caudate nucleus and tail, the right cholinergic basal forebrain, hippocampus, left more than right insula, right parahippocampal gyrus, amygdala, putamen, anterior-intralaminar- lateral posterior- mediodorsal—pulvinar—ventral nuclei of thalamus, metathalamus (including right more than left lateral geniculate nuclei [LGN]), pons, midbrain (esp. tectum), and cerebellar hemispheres. Table 2 provides details on the main significant clusters, peak MNI coordinates, and the associated regions. Non-PVC analysis reiterates the topography of the correlated deficits observed in the PVC-corrected images but reveals a greater number of statistically significant voxels in the brainstem, pons, and midbrain (Supplementary section S2). Additional analyses were performed to investigate cholinergic loss in PSP patients compared with controls; these findings are presented in Supplementary sections. Furthermore, a regression analysis was conducted on nine PSP subjects, using their total PSP Rating Scale scores as the dependent variable. Sex, disease duration, and Levodopa Equivalent Daily Dose (LEDD) were included as covariates. As with the primary analyses, all additional analyses were corrected for multiple comparisons using FWE cluster-wise correction. Only the significant clusters that survived correction are displayed in Supplementary sections S3, S4, respectively.

**Table 2:** Significant MRPI 2.0 associated reduced [^18^F]-FEOBV binding clusters using SPM voxel-based morphometry analysis corrected for multiple comparisons using cluster-level Family Wise Error correction (FWE; *p* < 0.01), showing the peak voxel location, *t*-values, and associated brain regions for each cluster.

Cluster (voxels)	Peak MNI coordinates			BA	Peak *t-*value	Peak voxel location	Associated brain regions
	X	Y	Z				
58	−38	−76	−46		3.70	Left crus II of cerebellar hemisphere	Left crus II of cerebellar hemisphere
139	−22	−88	−34		4.20	Left crus II of cerebellar hemisphere	Left crus I and II of cerebellar hemisphere
4,062	0	−30	−2	11,13,22,24,25,28,32,34,38,47	6.91	Midbrain	Right Amygdala Right more than left Caudate nuclei and tails Left lobule III, IV, and V of cerebellar hemisphere Right more than left hippocampus Right Insula Bilateral lingual Right nucleus accumbens Right orbital frontal cortex Left olfactory cortex Right Para-hippocampal gyrus Right Putamen Right Gyrus Rectus Right superior temporal pole Lobule I, II and III of vermis Right more than left anterior nuclei of thalamus Right more than left intralaminar nuclei of thalamus Right LGN Bilateral lateral posterior nuclei of thalamus Bilateral mediodorsal nuclei of thalamus Bilateral pulvinar nuclei of thalamus Right more than left ventral nuclei of thalamus Pons Midbrain Right basal forebrain
610	−42	6	−8	13, 22, 38, 47	4.57	Left insula	Left insula Left putamen Left superior temporal pole Left superior temporal gyrus Left posterior orbital frontal cortex Left Heschl gyrus
132	−38	−25	−16	36	4.52	Left fusiform	Left inferior temporal gyrus Left hippocampus Left fusiform gyrus
177	−42	24	44	8,9	7.03	Left middle frontal gyrus	Left middle frontal gyrus

### *Post hoc* univariate regression analyses

**Figure 2:**
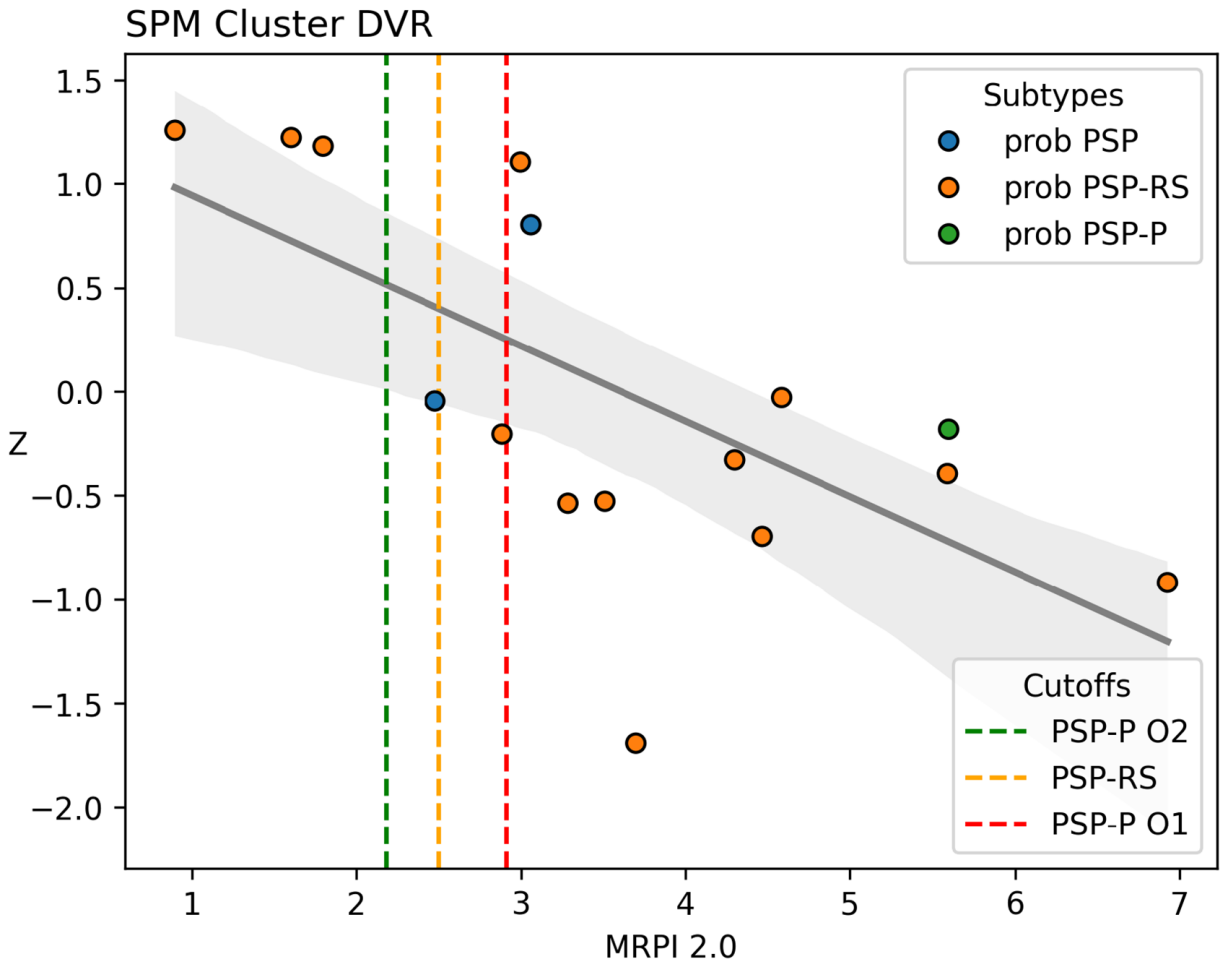
Scatterplot with line of best fit for association between MRPI 2.0 scores and SPM significant voxel clusters DVR as obtained from the primary voxel-wise analysis. DVR values on the Y-axis were residualized for LEDD and disease duration to better reflect the partial correlation between the two primary variables of interest. Dashed lines represent MRPI 2.0 cutoffs for different subtypes of PSP. Green dashed vertical line represents the PSP-P O2 level cutoff (>2.18), orange represents the PSP-RS cutoff (>2.5), and red represents the PSP-P O1 level cutoff (>2.91).

*Post hoc* multiple regression analysis revealed that 59.3% of the variance in [^18^F]-FEOBV uptake within the SPM significant voxel clusters (as observed in the voxel-wise analysis) was explained by MRPI 2.0 scores. There was a statistically significant negative association between MRPI 2.0 scores and SPM voxel clusters DVR (*β* = −0.8 [−1.226, −0.373], *p* = 0.002), whereas no independent association was observed for LEDD (*β* = 0.032 [−0.413, 0.478], *p* = 0.876), disease duration (*β* = 0.187 [−0.267, 0.640], *p* = 0.384), and sex (*β* = −0.099 [−0.963, 0.764], *p* = 0.805). The scatterplot for correlation between MRPI 2.0 scores and SPM cluster DVR is presented in Figure 2.

**Figure 3:**
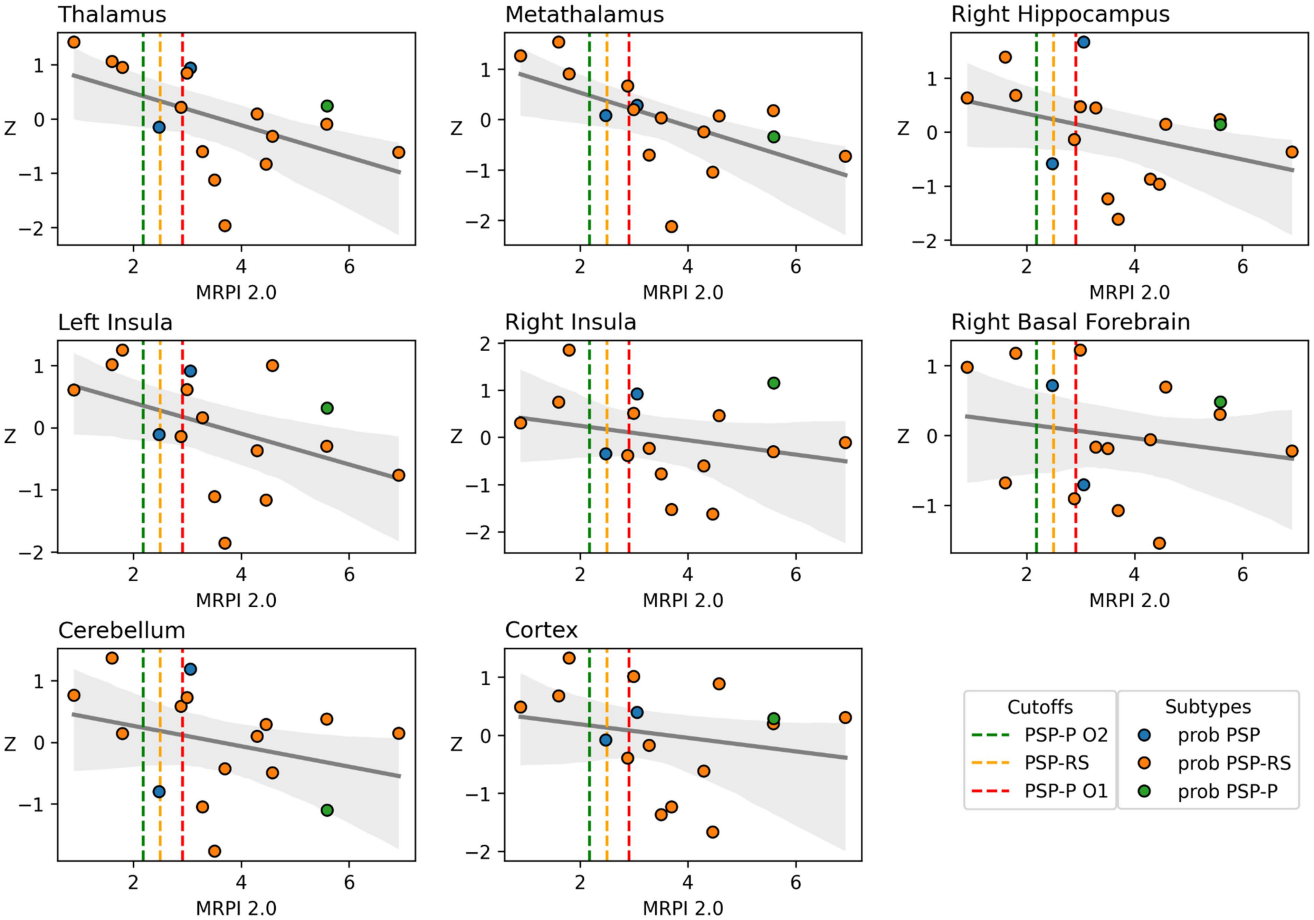
Scatterplot with line of best fit for association between MRPI 2.0 scores and PET-native space regional mean DVR values. DVR values on the Y-axis were residualized for LEDD and disease duration to better reflect partial correlations between the two primary variables of interest. Dashed lines represent MRPI 2.0 cutoffs for different subtypes of PSP. Green dashed vertical line represents the PSP-P O2 level cutoff (>2.18), orange represents the PSP-RS cutoff (>2.5), and red represents the PSP-P O1 level cutoff (>2.91). Individual data points are colored based on PSP participant subtype identification. All PSP-P and all but 3 PSP-RS subtype patients were above their respective MRPI 2.0 cutoff. A single probable PSP patient was above all three potential cutoffs, while the other probable PSP patient fell above the PSP-P O2 and just below the PSP-RS cutoff.

*Post hoc* multiple regression analyses of native space PET VOIs are presented in Table 3. The greatest explained variance in [^18^F]-FEOBV uptake was in the thalamus, the only region examined where the association between MRPI 2.0 scores and cholinergic terminal density was statistically significant independently of LEDD and disease duration. Scatterplots for the examined regional associations are presented in Figure 3.

**Table 3:** Multivariate volume-of-interest (VOI) based correlation models between regional [^18^F]-FEOBV uptake and MRPI 2.0 values after adjustment for LEDD, disease duration (years from symptom onset), and sex.

Domain	LEDD (mg)	Disease duration (years)	Sex (female)	MRPI 2.0	*R* ^2^	*P*
Thalamus	−0.111 [−0.7, 0.478]	0.288 [−0.311, 0.888]	−0.335 [−1.476, 0.806]	−0.65* [−1.213, −0.087]	0.478	2.75e-02*
Metathalamus	−0.141 [−0.672, 0.39]	0.314 [−0.227, 0.854]	−0.526 [−1.555, 0.502]	−0.732* [−1.239, −0.224]	0.576	8.9e-03*
Left insula	−0.099 [−0.728, 0.531]	0.151 [−0.489, 0.792]	0.185 [−1.035, 1.405]	−0.546 [−1.148, 0.056]	0.404	7.11e-02
Right insula	0.068 [−0.658, 0.794]	−0.039 [−0.777, 0.7]	0.401 [−1.005, 1.807]	−0.337 [−1.031, 0.357]	0.208	3.08e-01
Right hippocampus	0.218 [−0.449, 0.884]	0.062 [−0.616, 0.74]	0.167 [−1.125, 1.459]	−0.467 [−1.105, 0.171]	0.332	1.35e-01
Right basal forebrain	−0.025 [−0.692, 0.643]	0.497 [−0.183, 1.176]	0.044 [−1.249, 1.337]	−0.221 [−0.859, 0.417]	0.33	4.62e-01
Cerebellum cortex	0.132 [−0.537, 0.8]	0.185 [−0.495, 0.865]	0.36 [−0.935, 1.655]	−0.365 [−1.004, 0.274]	0.329	2.35e-01
Cerebral cortex	0.096 [−0.591, 0.784]	−0.232 [−0.932, 0.468]	0.751 [−0.582, 2.084]	−0.258 [−0.916, 0.4]	0.288	4.07e-01

Standardized regression coefficients with 95% confidence intervals are presented for both covariate measures (LEDD and disease duration) and the primary explanatory variable (MRPI 2.0). Total model coefficient of determination (*R^2^*) and MRPI 2.0 regression coefficient *p*-values are presented. Statistically significant model coefficients (and the corresponding *P*-value for the MRPI 2.0 coefficient) are marked with an asterisk (*).

### *Post hoc* regional volume analysis

Correlation between normalized regional brain volumes and MRPI 2.0 index are presented in Supplementary section S5. The overall pattern of atrophy associated with MRPI 2.0 appears to primarily affect subcortical regions, which is consistent with our findings of MRPI 2.0 related cholinergic losses are primarily in subcortical regions.

## Discussion

Our investigation aimed to delineate the relationship between regional MRPI 2.0 and regional subcortical and cortical [^18^F]-FEOBV uptake within a PSP cohort. Using SPM12 voxel-wise and *post hoc* multiple regression analysis, we observed robust correlations between these two distinct imaging markers across various brain regions, primarily subcortical structures. These relationships largely persisted even after accounting for confounding factors like sex, LEDD, and disease duration. This co-occurrence provides novel insights into the complex interplay between midbrain pathology, as captured by MRPI 2.0, and cholinergic systems dysfunction, a key contributor to cognitive and motor deficits in PSP.

MRPI 2.0 is established as a valuable imaging biomarker for differentiation of PSP from other atypical parkinsonian syndromes and PD, reflecting PSP-specific structural brain atrophy, namely changes in the brainstem, cerebellar peduncles, and size of the lateral and third ventricles (Quattrone et al., 2018; Cunningham et al., 2025; Quattrone et al., 2023). Tau pathology in PSP initially accumulates largely in subcortical and brainstem regions (Kovacs et al., 2020). These brainstem regions are not only central to PSP’s characteristic motor and ocular (VSGP) manifestations but also house crucial cholinergic projection neurons that modulate motor control (Gut and Mena-Segovia, 2019)—including the pedunculopontine nucleus (PPN) and the laterodorsal tegmental nucleus (LDT)—projecting to the thalamus and medial and limbic striatal regions (nucleus accumbens) (Dautan et al., 2014; Dautan et al., 2016). Neural degeneration in PSP is noteworthy in that it primarily affects cholinergic neurons in the midbrain early in the disease progression, significantly altering their projections and likely contributing to postural instability, and falls (Hirsch et al., 1987; Gut and Mena-Segovia, 2019). Accordingly, the strongest correlation with MRPI 2.0 was observed in subcortical structures innervated by PPN and LDT.

### Cholinergic system degeneration is likely a significant contributor to PSP pathophysiology

Within the organization of brain cholinergic systems, MRPI 2.0 values are expected to most strongly reflect changes in the perikarya and projections of cholinergic neurons located around the mesopontine junction. These neurons innervate the brainstem, thalamus, and basal ganglia. Consistent with this prediction, the voxel-based analysis identified correlated deficits in the thalamus, midbrain, and pons. In the complementary VOI-based analysis, correlated thalamic deficits were significant. Correlated deficits in other regions are harder to explain based on mesopontine cholinergic projection neuron degeneration. Striatal cholinergic terminals mainly originate from striatal cholinergic interneurons and cerebellar cortical cholinergic terminals are thought to originate in cholinergic medial vestibular neurons whose projections likely traverse the inferior cerebellar peduncle. The correlated basal forebrain deficits may reflect loss of mesopontine cholinergic afferents, but are more likely to reflect VAChT expression deficits in cholinergic basal forebrain neurons. Therefore, while MRPI 2.0 is most closely correlated with mesopontine cholinergic neuron degeneration, it may also reflect broader changes in brain cholinergic systems in PSP.

Mesopontine cholinergic neuron degeneration manifests early in disease progression, likely contributing to postural instability, falls, and executive dysfunction (Hirsch et al., 1987; Gut and Mena-Segovia, 2019). Previous immunohistochemical analysis revealed that mesopontine choline acetyltransferase levels are significantly reduced in PSP (Kasashima and Oda, 2003; Ruberg et al., 1985). Post-mortem studies indicate reduction of up to 60% of mesopontine cholinergic neurons in the PPN (Warren et al., 2005), with tau-positive neurons present (Eser et al., 2018). Correlating with mesopontine cholinergic projection neuron degeneration, thalamic acetylcholinesterase (AChE) activity is lower in PSP patients compared to those with PD (Gilman et al., 2010). Our results suggest that the pathology captured by MRPI 2.0 may, in part, reflect the degeneration of these cholinergic neurons and their projection pathways.

Deficits of other cholinergic systems are likely important in PSP. The nucleus basalis of Meynert (NBM), part of the basal forebrain, is the primary source of cholinergic innervation to the cerebral cortex (Kanel et al., 2022; Chakraborty et al., 2024; Crowley et al., 2024). Degeneration of basal forebrain projections account for the cortical cholinergic deficits impacting cognition, such as executive dysfunction (Chakraborty et al., 2024; Crowley et al., 2024). It is notable that, despite being correlated with the right cholinergic forebrain, only very limited neocortical regions were significant in the analysis. This suggests that MRPI 2.0 mainly correlates with subcortical cholinergic system changes in PSP. Cholinergic denervation found in subcortical areas such as the basal forebrain, caudate, putamen, thalamus, and brainstem nuclei like the pons and midbrain, aligns with the neuropathological hallmarks of PSP (Looi et al., 2011; Schulz et al., 1999; Cordato et al., 2002). The present findings suggest that the atrophy reflected by higher MRPI 2.0 scores, may be closely related to the pathological processes associated with subcortical cholinergic terminal loss.

We report novel findings of right-hemisphere-predominant cholinergic losses in PSP (affecting the caudate, basal forebrain, limbic regions, and metathalamus) that correlate with MRPI 2.0 values, but further validation is needed. We previously observed predominant right hemispheric, including caudate nucleus and right thalamic complex cholinergic losses in patients with PD with falls (Bohnen et al., 2019; Vossel et al., 2014). Note that the right hemisphere may be dominant for vestibular and ventral attention network functions that may associate with falls (Dieterich et al., 2003). Cholinergic thalamic afferents have key functions in multisensory processing, bottom-up signal detection, and postural control in PD (Kim et al., 2017; Müller et al., 2013) and most likely also in PSP. Left-lateralized findings, however, were also observed in the insula and cerebellum of PSP patients in relation to MRPI 2.0 index, suggesting a more complex assemblage of lateralized cholinergic deficits which would have to be disentangled by future studies.

Study limitations include the relatively small N of this convenience sample. The sample size likely reduced power to detect significant associations, perhaps explaining why only the thalamic association was significant in the VOI-based analysis. Additional limitations of this cross-sectional and correlational study include inability to address pathologic processes. While our study demonstrates significant correlations, the nature of underlying pathogenic processes remains unknown. Primary degeneration of cholinergic neurons in parallel with other structural changes is a plausible explanation for association between MRPI 2.0 and regional cholinergic deficits. Alternatively, cholinergic deficits could be secondary to other PSP-related pathologies. Widespread white matter damage, for example, potentially driven by tau deposition or neuroinflammation, could contribute to the degeneration of cholinergic terminals/axons and subsequent perikaryal degeneration. Future research should include longitudinal designs and larger cohorts, including various PSP subtypes, to further validate these findings. Future studies exploring the specific white matter tracts impacted by PSP using advanced diffusion imaging techniques in conjunction with cholinergic PET imaging could address the relationship between white matter changes and regional cholinergic denervation.

In conclusion, we describe significant correlations between higher MRPI 2.0 scores and characteristic cholinergic terminal deficits in PSP, especially in the thalamus. These findings reinforce the value of MRPI 2.0 as an accessible MRI biomarker for PSP and suggest that it reflects the integrity of the critical cholinergic systems in this devastating tauopathy. Moreover, this integrated imaging approach presents a promising pathway for enhancing early and differential diagnosis, monitoring disease progression, and guiding the development of targeted therapies for PSP.

## Data Availability

The raw data supporting the conclusions of this article will be made available by the authors, without undue reservation.
