# Supplementary material for: Brain atrophy and cholinergic denervation in progressive supranuclear palsy: an MRI and [18F]-FEOBV PET study
Source: Front Neurosci. 2025 Dec 3;19:1695541. doi: 10.3389/fnins.2025.1695541 (PMC12712803; doi:10.3389/fnins.2025.1695541)

**Supplementary Materials**

*S1. Magnetic Resonance Parkinsonism Index (MRPI) Calculation*

MRPI is a composite of three ratio measures. The first ratio is the cross-sectional area of the pons to the cross-sectional area of the midbrain on the midsagittal slice:


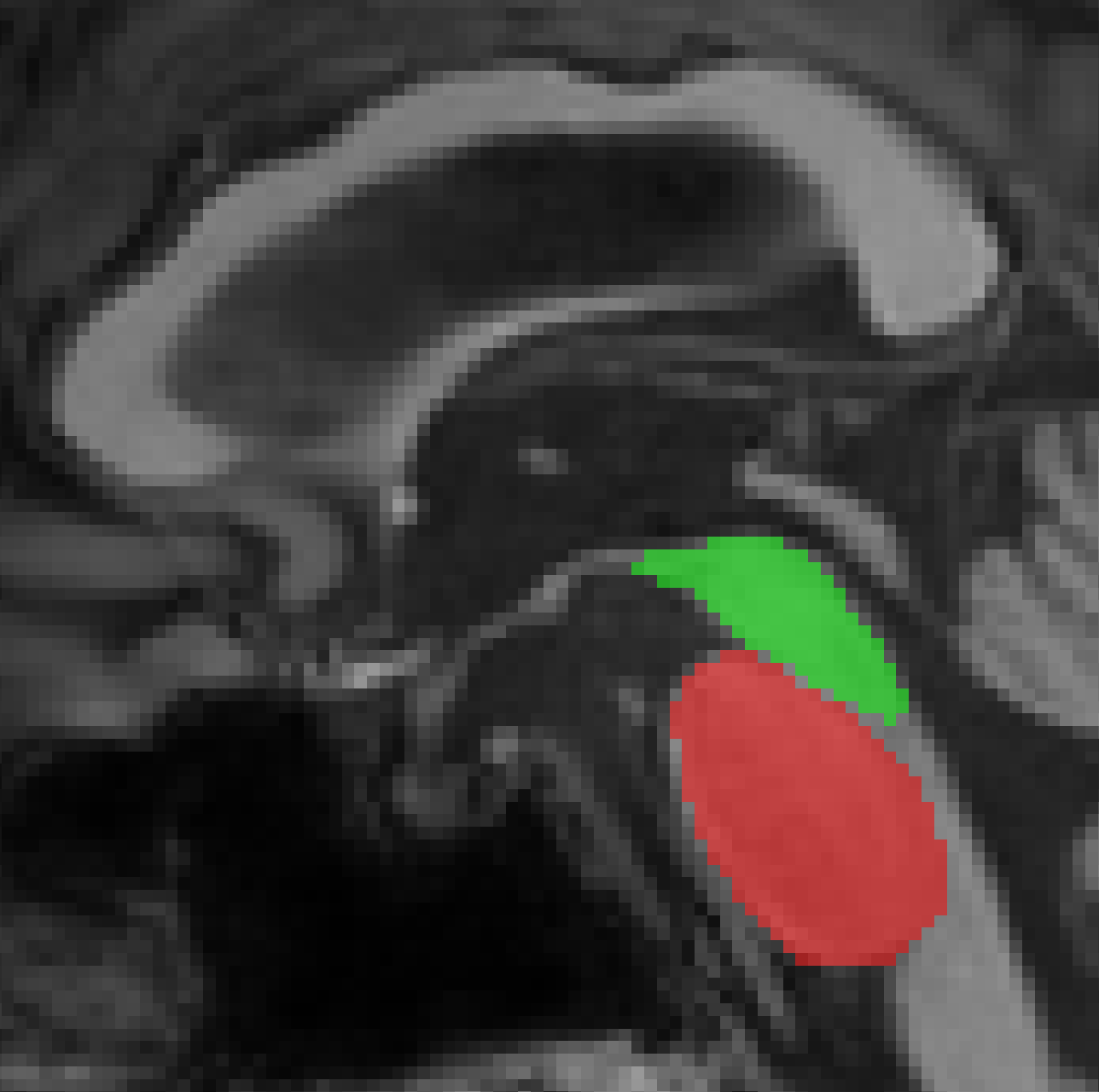


The second ratio is the average width of the left and right middle cerebellar peduncle (left image) to average width of the left and right superior cerebellar peduncle (right image):


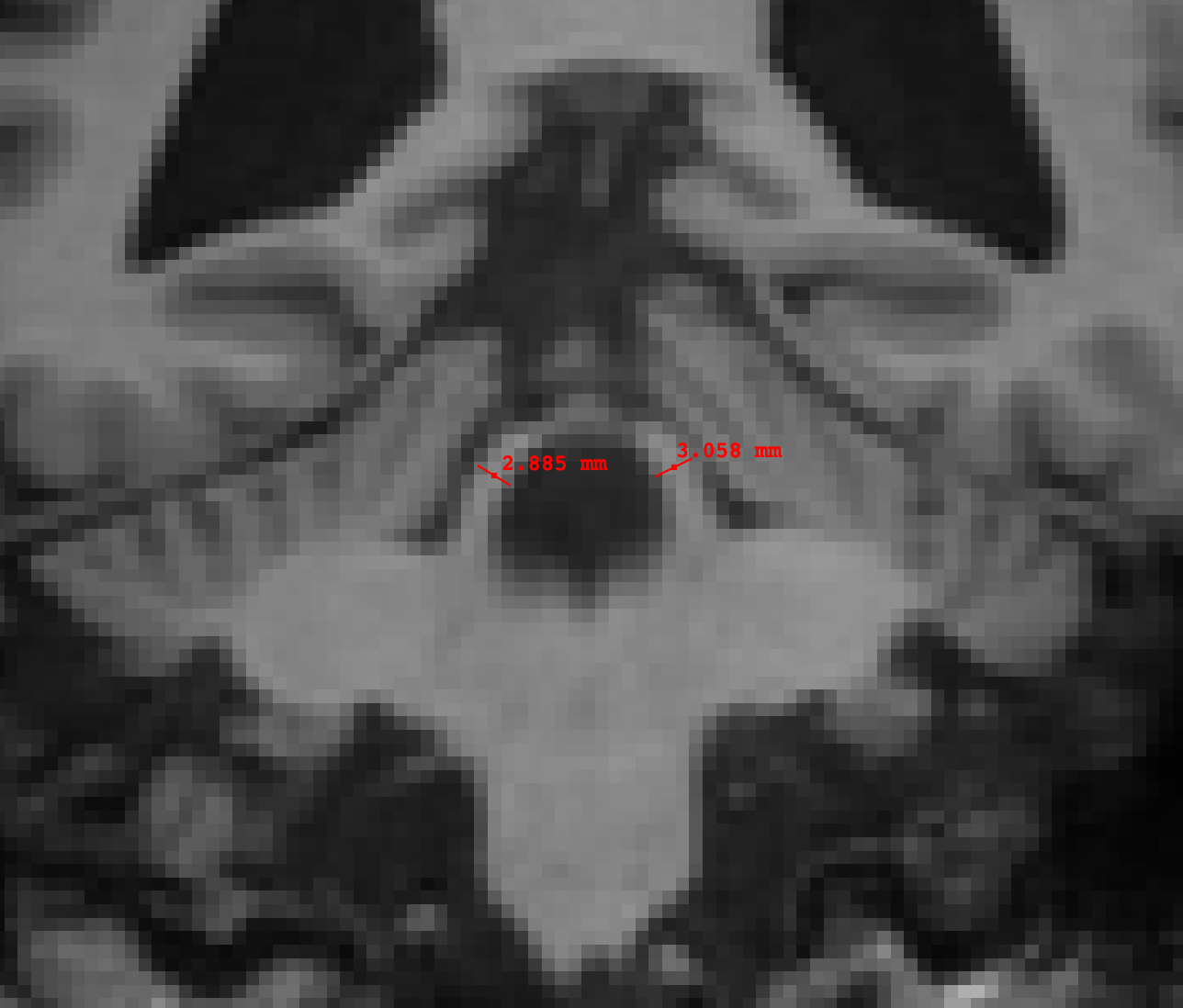

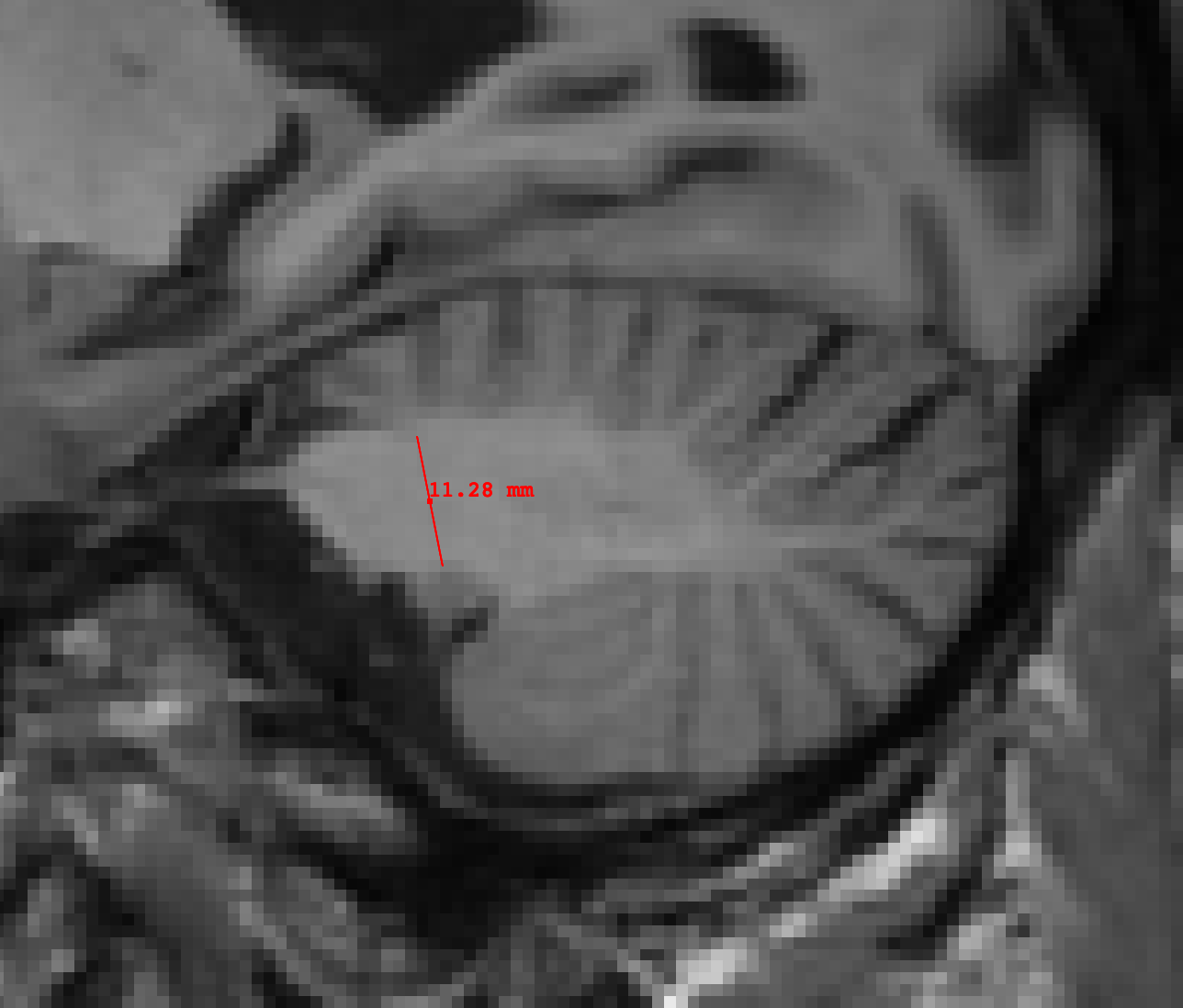


The third ratio (specific to MRPI 2.0) is the mean width of the third ventricle (derived from three measurements; top image) to the maximal length between the frontal horns of the lateral ventricles (bottom image):


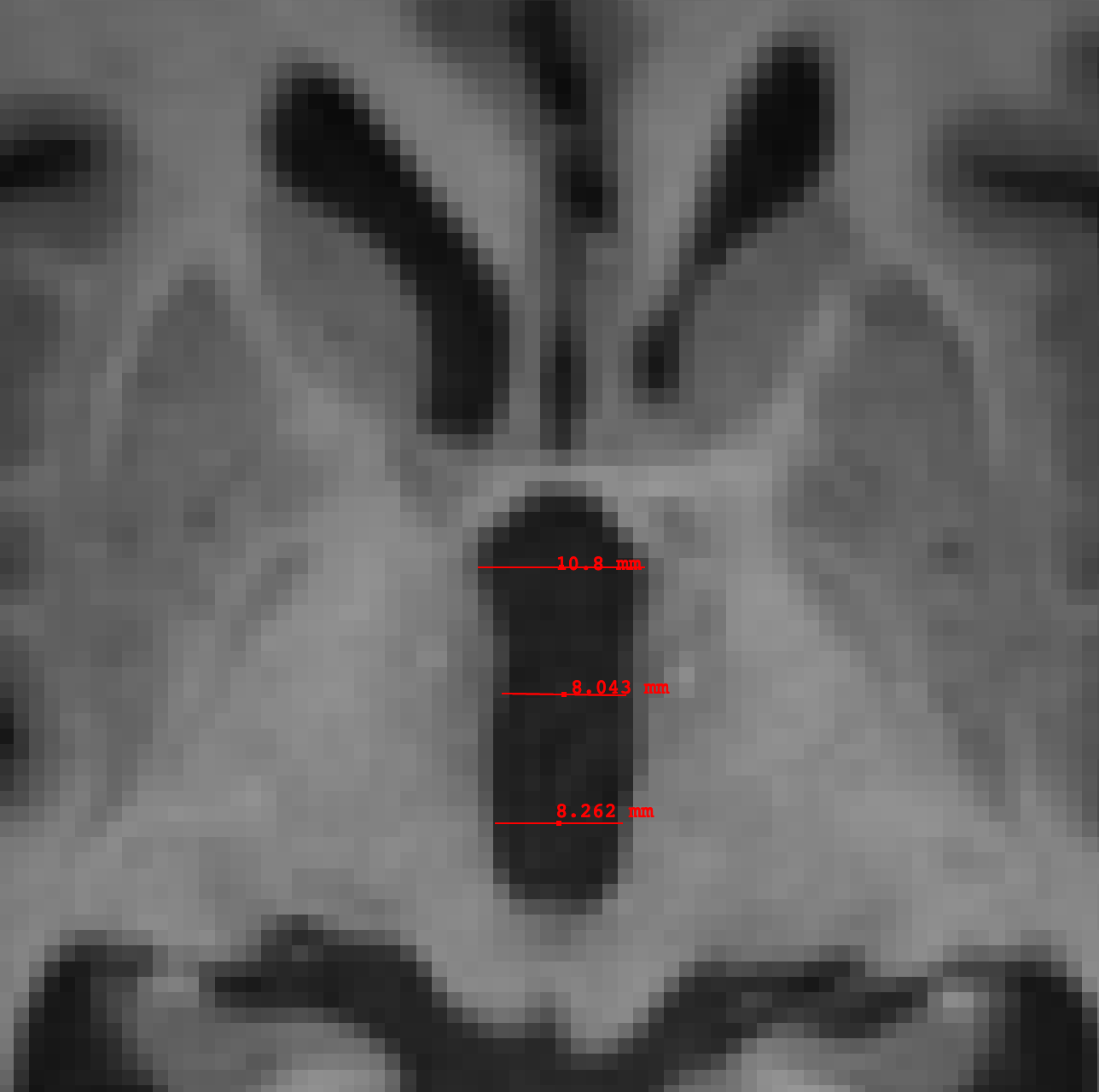


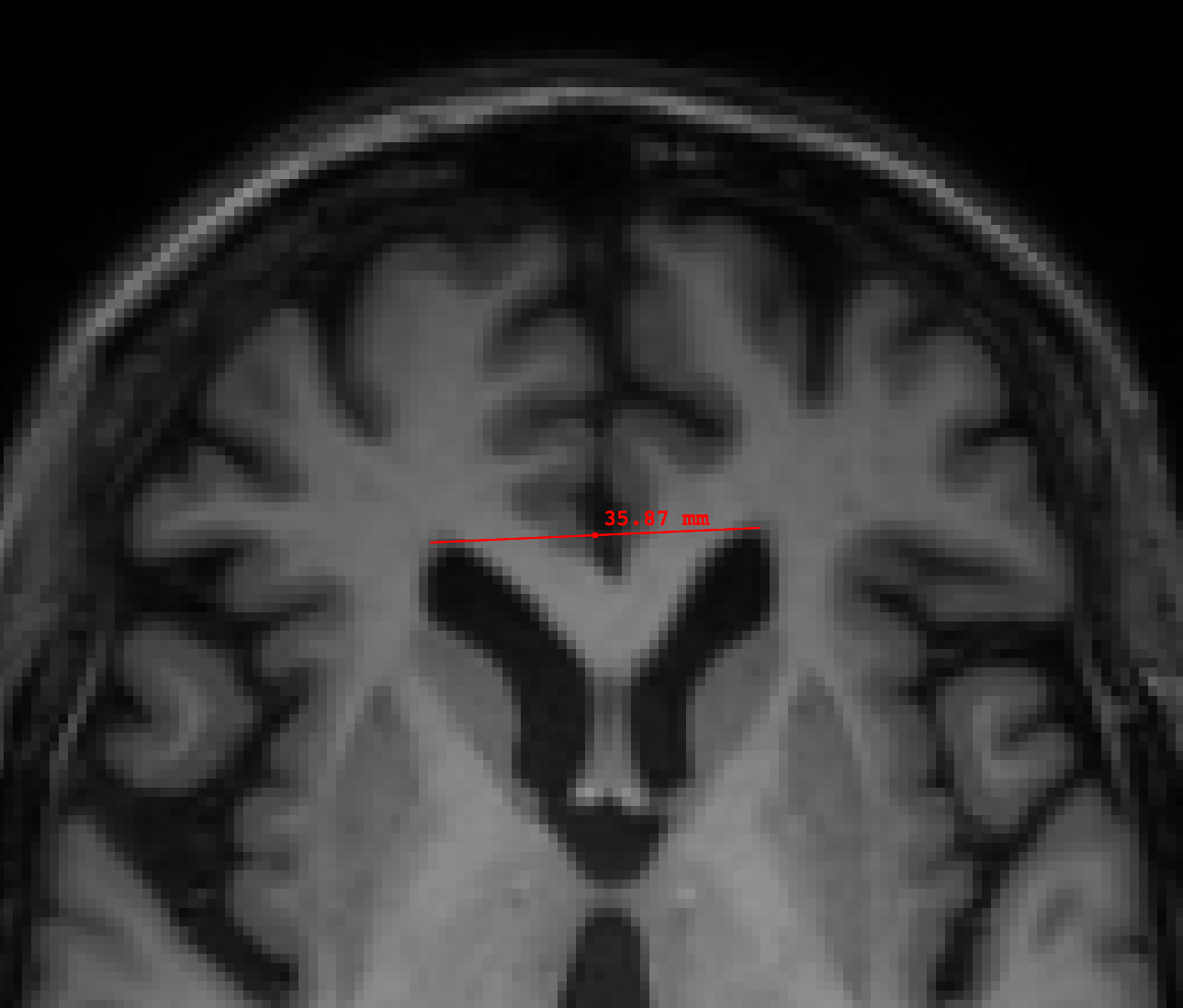


The final resulting measure is calculated as a product of these three ratios.

*S2. Voxel-wise correlation analysis on images without partial volume correction (PVC)*


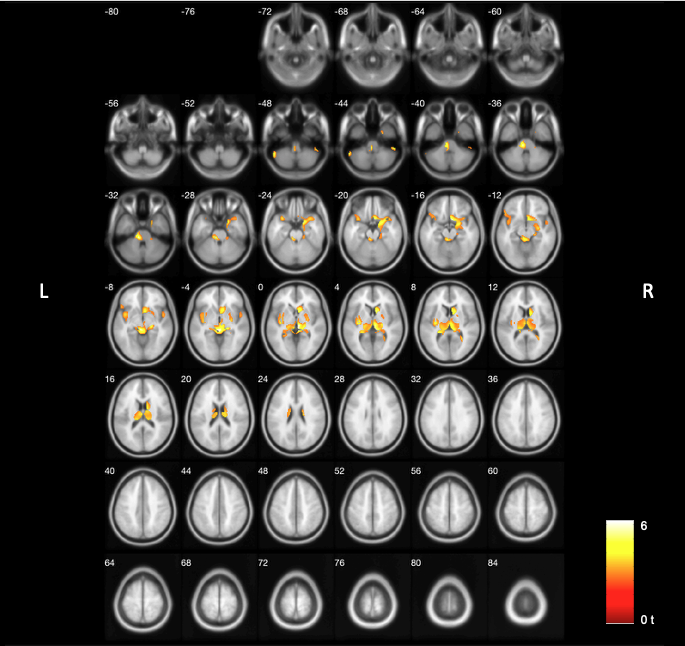


*S3. A voxel-wise analysis was conducted to compare patients with PSP to 15 healthy control (mean age 70.1 ± 6.45; 8 males/7 females). The results were evaluated with a threshold of uncorrected p < 0.01 and were corrected for multiple comparisons using cluster-wise FWE correction. Age and sex were included as covariates in the analysis. The clusters that survived the multiple-comparison correction are presented, highlighting regions with reduced cholinergic binding in PSP compared to healthy controls.*


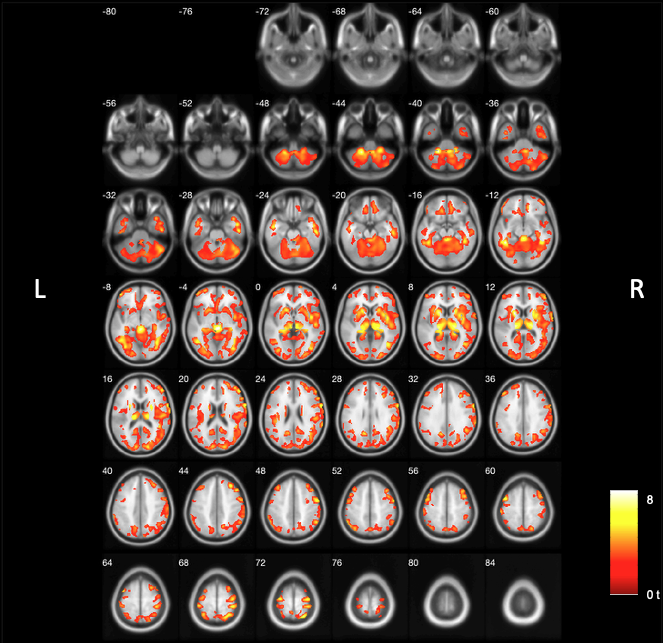


*S4.* A Voxel-wise regression analysis of *PVC corrected [18F]-FEOBV PET images revealed clusters of reduced cholinergic binding correlated with PSP Rating Scales on 9 PSP subjects*. The results were evaluated with a threshold of uncorrected p < 0.01 and were corrected for multiple comparisons using cluster-wise FWE correction. Disease duration, LEDD, and sex were included as covariates in the analysis. The clusters that survived the multiple-comparison correction are presented, showing regions with reduced cholinergic binding in PSP compared to healthy controls.

**
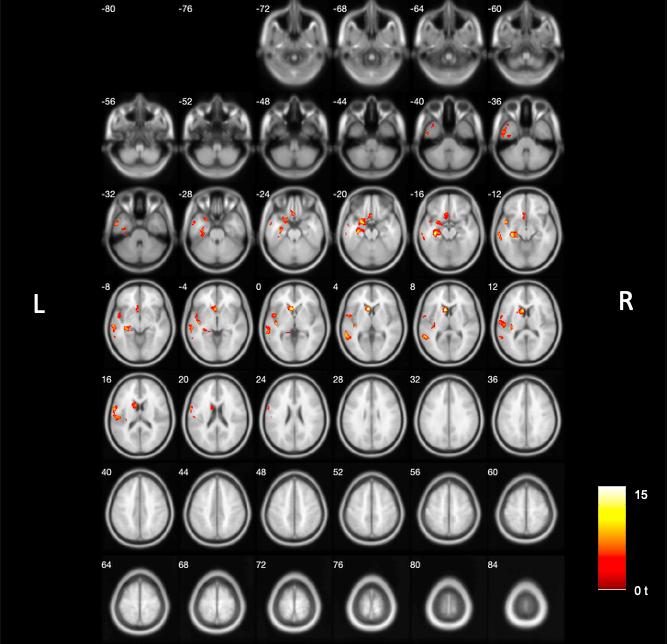
**

*S5. Regional volume univariate correlation topography with MRPI 2.0 index*


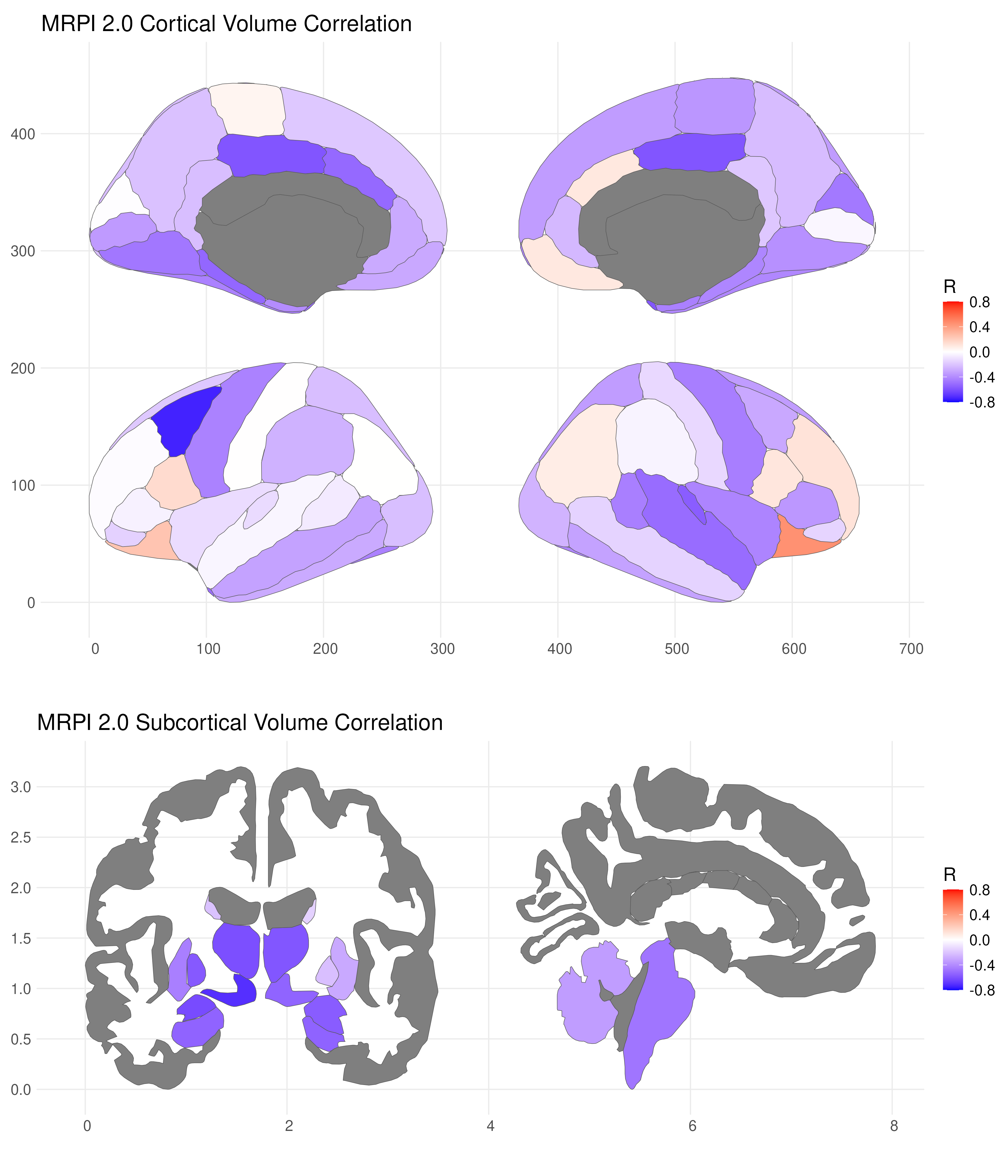

Supplement: Supplementary file 1 [file Data_Sheet_1.docx]
